# Microbial and genetically engineered oils as replacements for fish oil in aquaculture feeds

**DOI:** 10.1007/s10529-017-2402-6

**Published:** 2017-07-18

**Authors:** M. Sprague, M. B. Betancor, D. R. Tocher

**Affiliations:** Institute of Aquaculture, Faculty of Natural Sciences, University of Stirling, Stirling, FK9 4LA Scotland, UK

**Keywords:** Alternative n-3 LC-PUFA sources, Aquaculture, EPA and DHA, Farmed Fish, Human health, Oils from transgenic plants, Polyunsaturated fatty acids

## Abstract

As the global population grows more of our fish and seafood are being farmed. Fish are the main dietary source of the omega-3 (n-3) long-chain polyunsaturated fatty acids (LC-PUFA), eicosapentaenoic (EPA) and docosahexaenoic (DHA) acids, but these cannot be produced in sufficient quantities as are now required for human health. Farmed fish have traditionally been fed a diet consisting of fishmeal and fish oil, rich in n-3 LC-PUFA. However, the increase in global aquaculture production has resulted in these finite and limited marine ingredients being replaced with sustainable alternatives of terrestrial origin that are devoid of n-3 LC-PUFA. Consequently, the nutritional value of the final product has been partially compromised with EPA and DHA levels both falling. Recent calls from the salmon industry for new sources of n-3 LC-PUFA have received significant commercial interest. Thus, this review explores the technologies being applied to produce *de novo* n-3 LC-PUFA sources, namely microalgae and genetically engineered oilseed crops, and how they may be used in aquafeeds to ensure that farmed fish remain a healthy component of the human diet.

## Introduction

The continual increase in the global population presents a significant challenge to food security, particularly with respect to providing an adequate supply of nutritious, safe and affordable high quality food. Seafood is a rich source of protein, vitamins and minerals as well as being the main dietary source of the omega-3 (n-3) long-chain polyunsaturated fatty acids (LC-PUFA), eicosapentaenoic (EPA; 20:5n-3) and docosahexaenoic (DHA; 22:6n-3) acids, that are conditionally essential for human health and development (Calder 2014; Tocher 2015). Indeed, consuming at least two portions of fish per week, of which one should be oily, is advised by global health authorities as a means of achieving a recommended daily intake of 250–1000 mg EPA + DHA in order to protect against cardiovascular and inflammatory diseases among other health benefits (SACN/COT 2004; GOED 2014). Nevertheless, the capacity of wild capture fisheries to satisfy the increased demand for seafood is limited and unsustainable.

Aquaculture, the farming of aquatic organisms, is currently the fastest growing animal protein-food producing sector supplying over 50% of the world’s fish and seafood for human consumption (FAO 2016). However, aquafeeds have traditionally relied upon the inclusion of marine ingredients, fishmeal and fish oil, themselves sourced from wild fisheries to supply essential nutrients as well as the high EPA and DHA levels typically associated with farmed carnivorous marine fish species such as Atlantic salmon (*Salmo salar*). As the industry has grown to meet consumer demands, these finite marine ingredients have gradually been replaced in aquafeeds with alternatives, primarily of terrestrial agricultural origin. Marine ingredients in Norwegian salmon feeds, for instance, decreased from 90% to around 30% inclusion between 1990 and 2013 (Ytrestøyl et al. 2015). The replacement of the marine protein source, fishmeal, has generally been accomplished through the use of plant-based protein sources such as soy products, although these can possess negative traits such as amino acid imbalances or the presence of anti-nutritional factors, and are also susceptible to fluctuations in price (Gatlin et al. 2007). Nonetheless, reducing the reliance on marine protein sources differs greatly from that of reducing marine oil use.

Fish and plant oils both contain n-3 polyunsaturated fatty acids (PUFA) but terrestrial plants only contain the shorter-chain type such as α-linolenic acid (ALA; 18:3n-3) and are completely devoid of any EPA and DHA. As with humans, coldwater marine finfish are inefficient at converting ALA, the metabolic precursor, into appreciable levels of the beneficial EPA and DHA and, therefore, these must be supplied in the diet (Tocher 2015). Thus, the increasing use of vegetable oil as a replacement for fish oil in aquafeeds has subsequently resulted in the decline of the beneficial n-3 LC-PUFA in farmed fish, particularly salmon, thereby reducing the nutritional value to the final consumer and questioning the current dietary guidelines with respect to fish intake (de Roos et al. 2017; Sprague et al. 2016). With gaps in the actual versus recommended intake of EPA and DHA for the majority of the world’s population (Stark et al. 2016), and also in the supply and demand for n-3 LC-PUFA (Naylor et al. 2009; Tocher 2015), there is therefore a pressing need for novel *de novo* sources of n-3 LC-PUFA. Indeed, the Global Salmon Initiative has invited commercial organisations to supply its members with up to 200,000 tons annually of novel omega-3 rich oils to support the sustainable use of marine oils in aquafeeds (GSI 2015). This review explores the recent commercial interest by both agri-biotechnology and bioengineering companies and considers the main options that are likely to complement fish oil use as future sources of EPA and DHA in aquafeeds.

### Other marine sources

As n-3 LC-PUFA are largely derived from the marine environment it would therefore appear logical that the focus on alternative sources begins there. Both commercial and scientific interests have centred on lower trophic organisms with potential candidates such as Antarctic krill (*Euphasia superba*) and calanoid copepods (*Calanus finmarchicus*) already investigated as sources of n-3 LC-PUFA for use in salmonid feeds (Olsen et al. 2004, 2006). Although several species of krill have been fished, the main current large-scale fisheries are of Antarctic krill from the Southern Ocean as well as Pacific krill (*Euphasia pacifica*) off the coasts of Japan and Canada (Olsen et al. 2011). The primary commercial drivers for the current krill fishery are nutritional supplements for direct human consumption due to krill oil having a uniquely high content of phospholipid-bound n-3 LC-PUFA, which are thought to be more bioavailable, as well as being involved in regulating more metabolic pathways than the triacylglycerol-bound EPA and DHA found in fish oils (Ulven and Holven 2015). The by-product, krill meal is currently used in small amounts as a speciality ingredient in niche aquaculture feeds, primarily based on beneficial palatability/feed attractant, antioxidant and immune enhancing properties (Katevas 2014). Copepods accumulate high levels of lipids of up to 50–70% of body dry weight that are of interest to aquaculture (Falk-Petersen et al. 2009), although only small-exploratory-scale harvesting of *Calanus* has occurred to date (Olsen et al. 2011). Nevertheless, C-Feed AS (Trondheim, Norway) currently grow copepods on a commercial scale as live feeds for early developmental stages of marine fish, crustaceans and other marine organisms (C-Feed 2014). In addition to the above, there is also a growing interest in using underutilized sources such as fishery and aquaculture by-products and mesopelagic fish among other marine sources (Olsen et al. 2011). However, production volumes from all these sources are relatively low and, although apparently sustainably managed, concerns have been raised regarding the potential effects that harvesting down the trophic chain may have on higher trophic species that rely upon this food source (Hill et al. 2006).

### Microalgae and microbial sources

Microalgae, along with other single cell microbes, are the primary producers of n-3 LC-PUFA in the aquatic environment, providing a continual supply of EPA and DHA that is concentrated through the trophic food chain where there is limited capacity to synthesize these beneficial fatty acids. Accordingly, microbial sources offer a natural way of increasing the supply of n-3 LC-PUFA for farmed fish and have already been used by the aquaculture industry to enrich live feeds, rotifers and *Artemia*, in these fatty acids prior to feeding to larval fish (Barclay and Zeller 1996; Benemann 1992; Nichols et al. 1996; Lewis et al. 1998) as well as directly in formulated feeds for larval and juvenile fish (Eryalçin et al. 2013; Ganuza et al. 2008; Norambuena et al. 2015; Sarker et al. 2016). However, it is at later life stages (grow-out), where microalgae are not the natural food source and greater volumes of product are required, that microbial biotechnology can potentially deliver an alternative and promising approach to the traditional marine-derived ingredients for aquafeeds.

Several factors need to be taken into consideration when screening potential microbial sources for mass culture. Species and strain are both important with ideal candidates possessing key desirable characteristics such including a high n-3 LC-PUFA and oil contents, fast growth rate and the capacity for large-scale cost-effective production. Species that have high levels of EPA or DHA but lack the high lipid content required for enriching flesh of farmed fish for human nutrition may be suitable as alternative protein sources in aquafeeds. *Phaeodactylum tricornutum*, *Nannochloropsis* sp. and *Desmodesmus* sp. for example, have all been investigated as fishmeal replacements in salmon feeds with varying results (Gong et al. 2017; Sørensen et al. 2016), and may one day offer the possibility for the complete replacement of fishmeal and fish oil in aquafeeds as their n-3 LC-PUFA content could at least meet the essential fatty acid (EFA) requirements of the fish being farmed. Complete replacement of both fishmeal and fish oil has already been achieved using microalgae in juvenile Nile tilapia, *Oreochromis niloticus* (Sarker et al. 2016). Similarly, other microbial species may provide other benefits as feed additives in aquafeeds such as the freshwater green microalgae, *Haematoccocus pluvialis*, in replacing synthetic carotenoids in salmon feeds (Benemann 1992; Panis and Rosales Carreon 2016), although products originating from bacteria, derived from *Paracoccus carotinfaciens* and marketed as Panaferd-AX (Nippon Oil Corporation, Japan), and yeast, derived from *Phaffia rhodozyma*, are already used by the salmon industry. In addition to species, culture conditions (e.g. light, temperature, nutrient source) can also affect algal composition, including fatty acid profile, thereby complicating the ability to produce a product of consistent quality and/or in sufficient quantity (Glaude and Maxey 1994; Jiang et al. 1999; Hamilton et al. 2015). Due to these issues, culture of photoautotrophic microalgae in outdoor ponds or photobioreactors is currently insufficient to produce the high n-3 LC-PUFA biomasses required by global aquaculture. In contrast to photoautotrophic systems, heterotrophic production via fermentation, which utilizes sugars and other carbon sources for energy, may take only a few days and can therefore be tailored to meet market requirements.

The main driver for using heterotrophic microalgae as a source of n-3 LC-PUFA in aquaculture has arisen largely from the progression made in the more lucrative nutraceutical market, specifically the infant formula sector, which has the largest global market share for packaged EPA/DHA products other than fish (Packaged Facts 2012). Microbial species such as the marine diatom, *Crypthecodinium*, as well as the thraustochytrids, *Thraustochytrium*, *Ulkenia*, but especially *Schizochytrium* sp., have been identified due to their ease of cultivation under controlled conditions to produce high lipid biomasses rich in n-3 LC-PUFA and with high n-3:n-6 PUFA ratios (Lewis et al. 1999; Nichols et al. 2004; Ratledge 2005). However, one feature of *Schizochytrium* sp., and of thraustochytrids in general, is that they also produce significant amounts of n-6 docosapentaenoic acid (DPA; 22:5n-6), usually at a level of around 20% of the level of DHA produced, and this may increase the levels of n-6 DPA in the flesh of fish fed algal biomass (Kousoulaki et al. 2015, 2016; Sprague et al. 2015). However, n-6 DPA is considered a metabolically neutral fatty acid that has no overall impact on DHA uptake in humans (Nauroth et al. 2010; Ratledge 2005). Furthermore, the biomass grown under controlled heterotrophic fermentation is generally free from contaminants such as heavy metals, dioxins and polychlorinated biphenyls (Ratledge 2005), resulting in lower levels of these undesirables in both the feed and fillet flesh than in fish fed a standard marine fish oil diet (Sprague et al. 2015).

Miller et al. (2007) demonstrated that oil extracted from *Schizochytrium* biomass can be used at high inclusion levels to replace fish oil in the diets of salmon parr with no detriment to growth while also increasing flesh DHA levels. However, a major limitation to using algal oil in aquafeeds is that it can be expensive to extract from the biomass, compared to current fish oil prices. Once extracted, there are also additional difficulties related to protecting the LC-PUFA from oxidation as the natural antioxidants within the microalgae are no longer effective once the cell is ruptured during the extraction process (Miller et al. 2007; Winwood 2013). Carrier oils containing antioxidants, such as high oleic sunflower oil, may be used to stabilize the refined oil (Gray 2010). However, due to these issues, most commonly it has been the lipid-rich (generally ~40–60%) whole cell algal biomass that has been used directly in aquafeeds, although this itself can present problems particularly during the extrusion process when manufacturing the feeds. Carter et al. (2003), for example, replaced fish oil with a thraustochytrid biomass in juvenile salmon during the seawater transfer phase and found no difference in growth performance, although fish fed fish oil-based diets performed better under challenging conditions when presented with a *Vibrio anguillarum* bacterium. In a 12-week study in which post-smolts were grown from ~200 to 800 g and fed graded levels of *Schizochytrium* sp. biomass replacing fish oil, Kousoulaki et al. (2015) found that fish fed all inclusion levels performed well in terms of growth, health and fillet quality, including DHA retention particularly at 6% compared to 15% inclusion. In a similar study, Sprague et al. (2015) used a commercially available drum-dried *Schizochytrium* sp. biomass (DHAgold, formerly AquaGrow Gold; DSM Nutritional Products, Parsippany, NJ, USA) at 5.5 and 11% inclusion to replace fish oil in post-smolt salmon grown from 1500 to ~3000 g. Although salmon fed the 11% *Schizochytrium* biomass exhibited a minor, but significant, lower growth rate than fish fed a fish oil diet, flesh DHA levels were similar and higher than fish fed 5.5% algal biomass. Caution should always be applied when comparing studies, not only due to the different fish sizes/life-stages used in experiments, but also due to differences in the composition of control/reference diets used. For example, the reference diets used by Sprague et al. (2015) contained 27% fish oil as the sole dietary oil source, whereas, in the studies of Kousoulaki et al. (2015) and Carter et al. (2003), reference diets contained fish oil levels of 15 and 9.1%, respectively, in combination with vegetable oils.

Commercial salmon feeds currently generally incorporate blends of vegetable and fish oils to meet the nutritional demands of the fish being farmed, although compositions are continually evolving due to the pressures on marine sources, thereby altering n-3 LC-PUFA fillet levels (Ytrestøyl et al. 2015; Sprague et al. 2016). Thus, the fish farming industry must decide whether future sources are required simply to maintain EPA and DHA at current levels, where diets are mainly of vegetable origin with relatively low n-3 LC-PUFA contents, or whether the aim is to return to the historical levels obtained when diets contained high levels of fishmeal and fish oil and thus, much higher EPA and DHA contents. However, it is likely that both production volumes and the economics of microalgae production will ultimately be the determining factor in this choice. Nevertheless, *Schizochytrium* still offers potential as an alternative to fish oil, not only for salmon, but also for other commercially important species such as gilthead sea bream, *Sparus aurata* (Ganuza et al. 2008), channel catfish *Ictalurus punctatus* (Li et al. 2009), Nile tilapia (Sarker et al. 2016) and Pacific white shrimp, *Litopenaeus vannamei* (Wang et al. 2017) in which *Schizochytrium* products have been investigated.

Within the last year several algae-based and animal nutrition companies have announced novel product lines specifically aimed for use in aquaculture as sustainable alternatives to fish oil. This was largely stimulated by the Global Salmon Initiative’s call towards the end of 2015 for organizations to develop and produce 25,000 to 200,000 tons annually of novel oils with levels of n-3 LC-PUFA sufficient to support fish farming (GSI 2015). Archer Daniels Midland (ADM) Animal Nutrition (Quincy, IL, USA) for example, released a dried algae biomass, ‘DHA Natur’, with a DHA content of 17–20% as-is (ADM 2017). Alltech (Winchester, KY, USA) released their product, ‘ForPlus’, which may have been validated in salmon (Kousoulaki et al. 2015, 2016). The microalgal product has also been incorporated into their Coppens International, ‘NeoGreen’ fishmeal- and fish oil-free range of trout diets (Tsappis 2017). Meanwhile, TerraVia Holdings Inc. (formerly Solazyme; San Francisco, CA, USA) and Bunge Ltd. (White Plains, NY, USA) have jointly developed a whole algae biomass from *Schizochytrium* called ‘AlgaPrime DHA’ with a DHA content of ≥28% as-is, which is fermented using a sugarcane feedstock at a new production facility in Brazil (TerraVia Holdings, Inc. 2016). Moreover, the companies have an agreement to supply this product to the BioMar Group (Aarhus, Denmark), one of the three main global fish feed companies (Bunge 2017). These developments are a step in the right direction in terms of reversing the decline in n-3 LC-PUFA contents of farmed fish, especially salmon (Sprague et al. 2016).

All the above algal products, however, only provide one of the two beneficial n-3 LC-PUFA, DHA, and do not supply EPA. For example, Sprague et al. (2015) found that while no difference in DHA content was observed between salmon fed diets containing *Schizochytium* biomass and fish oil, the overall nutritional value to the human consumer, in terms of EPA + DHA, was lower due to the near absence of EPA in the algal feed. The fatty acids EPA and DHA exert a range of biological activities in humans, some overlapping but others distinct for each fatty acid (Calder 2014). Fish oil, therefore, remains the main source of marine-derived EPA at present, as EPA-producing algal sources have largely been of photosynthetic origin although attempts have been made to culture some species heterotrophically and optimize conditions to enhance biomass and lipid contents (Marudhupandi et al. 2016; Ratledge 2005). Nevertheless, a *Schizochytrium* strain/species with a minimum EPA and DHA content of 10 and 22% of total fatty acids, respectively, was reported previously (Gray 2010). Furthermore, DSM (Heerlen, Netherlands) and Evonik Industries AG (Essen, Germany) have recently formed a joint venture, *Veramaris*, coupling the former’s unique knowledge in cultivating marine organisms, especially *Schizochytrium*, with the latter’s expertise in developing industrial fermentation biotechnology, to produce an EPA + DHA-rich *Schizochytrium* algal oil for the animal feed industry. Full-scale production is anticipated in 2019 and will be situated in the USA, where the carbon source will be dextrose (corn syrup) obtained from local corn/maize production. The resultant extracted algal oil is expected to provide 15% of the total current annual demand by the salmon farming industry for EPA and DHA (DSM 2017). As previously mentioned, manufacturing a highly-concentrated oil from algal biomass has additional technical difficulties such as optimization of fermentation conditions to maximize n-3 LC-PUFA yield and upscaling to commercial production. An alternative approach may therefore be to genetically engineer microalgae to specifically optimize the production of the desired fatty acids, EPA and DHA.

### Transgenic sources

Although research has been undertaken to genetically modify microbes, including microalgae, in an attempt to produce or optimize n-3 LC-PUFA content (Hamilton et al. 2014, 2015), the main focus for bioengineering novel EPA- and/or DHA-rich oils as alternatives to fish oil has centred on oilseed crops. Terrestrial plants do not produce n-3 LC-PUFA because they lack the necessary genes (enzymes) required to elongate and desaturate shorter chain PUFA, namely ALA (18:3n-3), that can be abundant in some oilseed plants and, therefore, selective breeding cannot be used to develop new agricultural crop-based sources of EPA and DHA, making genetic modification (GM) the only option.

The biosynthesis of LC-PUFA in marine microalgae generally occurs through a series of aerobic elongation and desaturation reactions. The more common pathway involving a Δ6 desaturation of ALA followed by elongation to 20:4n-3 and then further desaturation by a Δ5 desaturase to produce EPA. Alternatively, ALA can be elongated to 20:3n-3 before undergoing sequential Δ8- and Δ5-desaturations to form EPA. DHA may then be synthesized by further elongation together with Δ4 desaturation (Harwood and Gushina 2009; Napier et al. 2015; Ruiz-Lopez et al. 2015). The genes involved in these pathways have all been identified and characterized in several microalgal species enabling their introduction into terrestrial oilseed plant hosts.

The two oilseed crops that have been viewed as potential platforms for engineering n-3 LC-PUFA are Camelina (*Camelina sativa*) and Canola (*Brassica napus* L.) with research primarily being led by the Agricultural Science Research Institute, Rothamsted Research (Harpenden, UK) and the Australian National Science Agency, the Commonwealth Scientific and Industrial Research Organisation (CSIRO, Australia). The successful accumulation of n-3 LC-PUFA into plant hosts has been primarily based upon work performed in the model species *Arabidopsis* (Petrie et al. 2012; Ruiz-Lopez et al. 2013). Ruiz-Lopez et al. (2014) then transferred this technology to Camelina as a host species with constructs consisting of five or seven marine microalgal genes for the purpose of engineering an EPA-only or an EPA + DHA oil, respectively. The authors reported an EPA content of 24% of total fatty acids in the EPA-only oil, whereas the EPA + DHA iteration gave an EPA and DHA content of 11 and 8% respectively. The levels of the substrate for EPA synthesis, ALA, and oleic acid (18:1n-9) were reduced in the transgenic plants. These n-3 LC-PUFA levels have also been replicated outside laboratory conditions when grown in the field, representing a viable alternative to fish oil use in aquafeeds (Usher et al. 2015). Similarly, using different constructs, Petrie et al. (2014) demonstrated significant levels of DHA production, of up to 12.4% of total fatty acids, in oil from transgenic Camelina, although EPA content was relatively low (0.8–3.3%) in comparison to that obtained by Ruiz-Lopez et al. (2014).

An alternative anaerobic pathway that can produce LC-PUFA, favoured by some bacteria and a few marine eukaryotes, involves polyketide synthase that can directly biosynthesize unsaturated fatty acids, including n-3 LC-PUFA, from C2 units supplied by malonyl-CoA without the need for additional desaturases (Harwood and Gushina 2009; Napier et al. 2015). Walsh et al. (2016) used this pathway to engineer Canola to produce an oil with EPA and DHA content of 0.7 and 3.7% of total fatty acids, respectively, equivalent to 600 mg in a 14 g serving. The same authors reported that by engineering the same PUFA synthase system in soybean, an oil containing 2.7% DHA and 1.5% EPA was achieved. While the levels of EPA and DHA produced using the polyketide approach were much lower than those demonstrated using the aerobic microalgal elongase/desaturase genes (Petrie et al. 2014; Ruiz-Lopez et al. 2014), it still represents a further option for the production of novel oils containing n-3 LC-PUFA.

To date, the only studies reporting the use of oils from n-3 LC-PUFA GM crops as ingredients in fish feeds have been those using oils from transgenic Camelina produced by Rothamsted Research. In the first of a series of trials, Betancor et al. (2015a, b) used the EPA-rich oil described by Ruiz-Lopez et al. (2014) in feeds for post-smolt salmon grown in seawater from ~80 to 200 g. No differences in growth performance, feed efficiency or survival were observed between dietary treatments. Furthermore, flesh of fish fed a diet containing the EPA-rich Camelina oil contained higher EPA levels than fish fed diets containing either the wild-type Camelina oil or fish oil while, as expected, flesh DHA levels were lower in fish fed both the transgenic and wild-type Camelina oil-based diets compared to fish fed the fish oil diet. In contrast, liver fatty acid profiles of fish fed the diet containing oil from transgenic Camelina showed higher levels of DHA and DPA (22:5n-3) as well as EPA compared to fish fed wild-type Camelina, indicating active biosynthesis of DHA from EPA, which was consistent with increased expression of hepatic desaturase (*fads*2*d*6 and *fads*2*d*5) and elongase (*elovl*2) genes in the fish fed the oil from GM Camelina.

In a subsequent study, also in salmon, Betancor et al. (2016a) investigated an oil containing around 6% each of EPA and DHA, obtained from a subsequent iteration of transgenic Camelina. Again, no differences in growth or health parameters were observed over the 12-week feeding trial, with fish growing from ~250 to ~540 g. Fish fed diet containing oil from transgenic Camelina demonstrated enhanced digestibility of EPA and DHA as well as higher levels of n-3 LC-PUFA in all tissues compared to fish fed a diet containing oil from wild-type Camelina. Furthermore, absolute amounts of n-3 LC-PUFA in fish fed the diet containing oil from transgenic Camelina were similar to those fed a diet containing fish oil. These initial studies utilized reference diets (termed fish oil diets above) that contained relatively high levels of fishmeal and fish oil, which represented “gold standard” feeds containing high levels of n-3 LC-PUFA. In a follow-up study, the EPA + DHA oil from transgenic Camelina was again investigated in salmon, but compared to a reference diet that reflected current commercial salmon feed formulations, with higher levels of plant meals and vegetable oil and lower levels of fishmeal and fish oil (Betancor et al. 2017). Following a 12-week feeding period, from an initial weight of ~120 g to a final weight of ~400 g, no significant differences between treatments were found in growth or health parameters. Moreover, fish fed the oil from transgenic Camelina contained almost double the n-3 LC-PUFA content than fish fed either the fish oil or wild-type Camelina oil feeds, thereby demonstrating the potential of the oils from GM crops to increase n-3 LC-PUFA levels in salmon. Further steps in the commercialisation will allow for the testing of these important n-3 LC-PUFA sources in harvest-sized salmon.

In addition to salmon, the EPA-only and EPA + DHA oils from transgenic Camelina have also been tested in another commercially important farmed marine species, gilthead sea bream (Betancor et al. 2016b). As in salmon, the GM-derived oils were found to be suitable alternatives to fish oil in sea bream diets. However, fish fed the EPA-only oil showed slightly reduced growth performance when compared to fish fed both the fish oil reference diet the and EPA + DHA Camelina oil, but similar growth to fish fed the wild-type Camelina oil. Although no obvious alteration in fish health was found in either histological or molecular observations the reduced growth in the EPA-only diet was suggested to be related to a dietary inbalance in fatty acid ratios, i.e. EPA/DHA or n-3/n-6, or specific fatty acids such as 20:4n-6. This additional study in sea bream, along with another study that demonstrated that the EPA-only oil was an effective source of bioavailable EPA in mice (Tejera et al. 2016), were further validations supporting the potential for commercialisation of these GM Camelina-derived oils, particularly as ingredients in feeds for farmed fish.

Production of the oils from GM oilseed crops should be straight forward as cultivation of the crops presents no specific agronomic difficulties and the processing technology and infrastructure is already in place for the extraction of oil from seed crops. The main issue in terms of potential production will simply be the volumes of oils produced using such technology. Napier et al. (2015) estimated that 200,000 ha of transgenic Camelina, less than 3% of the arable land (~7 million ha) presently used by Canada to grow oilseed crops, could theoretically produce 150,000 tonnes of oil equivalent to around 15% of the global harvest of marine oils.

At present, no commercial production of novel n-3 LC-PUFA oils, as defined as containing EPA and/or DHA, from GM oilseed crops has been reported. However, DuPont (Wilmington, DE, USA) developed a transgenic oleaginous yeast, *Yarrowia lipolytica*, to produce a high level EPA biomass through industrial-scale fermentation (Xi et al. 2015). The DuPont transgenic yeast biomass was used by AquaChile (Puerto Montt, Chile) to produce a niche product, Verlasso salmon, but recently the yeast biomass has apparently been replaced with a microalgal product, presumably a DHA-rich algal biomass (Verlasso 2017). The reason for this change is unclear, but it may be related to the fact that DHA has higher retention in salmon compared to EPA retention, which can be highly variable and even result in a net loss of EPA, especially when dietary DHA levels are low (Glencross et al. 2014).

Similarly, the Monsanto company (St Louis, MO, USA) has produced a GM soybean line that produces an oil containing stearidonic acid (SDA; 18:4n-3), a fatty acid that is more readily converted to EPA in humans than the ALA found in conventional plant sources (Harris et al. 2008). When fed to Atlantic salmon, SDA-rich oils, including the GM-soy oil, failed to elicit an increase in fillet EPA or DHA levels, although SDA was elongated to 20:4n-3 that, nonetheless, may be more easily converted to EPA in the human consumer compared to ALA found in the flesh of fish fed current formulations including high levels of rapeseed oil (Bell et al. 2006; Nanton et al. 2012; Tocher et al. 2006). Nevertheless, with the rapid development of novel EPA and DHA oils, it is unlikely that the GM-SDA-soy oil will be used to any large extent in aquafeeds, despite its approval for use in animal feeds in Europe (EFSA 2014), but may be included in foodstuffs for direct human consumption. It would therefore appear that the preferred new n-3 LC-PUFA sources, from both fish farming industry and human nutrition perspectives, will be oils that are rich in either DHA or, ideally, both EPA and DHA.

Collaboration between CSIRO, Nuseed (Nufarm Ltd., Melbourne, Australia) and the Australian Grains Research and Development Corporation (GRDC) has resulted in a DHA-rich Canola which, when grown, has the potential to produce a similar n-3 LC-PUFA yield to approximately 10,000 kg fish from just one hectare (Nuseed 2017). Company information suggests that the transgenic oil will be marketed for both use in aquafeed (Aquaterra) and human nutrition (Nutriterra) and, providing all the regulatory requirements are met, commercial production is expected to commence in Australia, Canada and the United States in either 2018 or 2019 (Nuseed 2017). Dow AgroSciences (Indianapolis, IN, USA) in association with DSM have developed both a soybean product containing 1.5 and 2.7% EPA and DHA, respectively, as well as the Canola oil containing 0.7% EPA and 3.7% DHA described by Walsh et al. (2016). Although the Canola oil has been field tested there is currently no information available as to when either product will be commercialized.

Another key player, the chemical company BASF SE (Ludwigshafen, Germany) and Cargill Inc. (Minnesota, MN, USA), the global food and agricultural company that also owns the fish feed producer Ewos, have partnered to develop a GM Canola producing a high n-3 LC-PUFA oil for use in aquafeeds by 2020 (Cargill 2016). No further information on the transgenic oil per se is available, but the biosynthetic pathway consisting of ten enzymes, used to engineer EPA and DHA in the transgenic Canola, has been demonstrated (Yilmaz et al. 2017). Calysta Inc. (Menlo Park, CA, USA) in partnership with CHAIN Biotech Ltd. (London, UK) are developing a GM *Methanococcus* microbe capable of converting methane gas into n-3 LC-PUFA during fermentation (Calysta 2016). The finished product is most likely to be high in protein and may feature as a fish meal replacement with the added benefit of being enriched in n-3 LC- PUFA.

While many of these GM products have been under development for many years, the Global Salmon Initiative’s call for novel oils high in n-3 LC-PUFA for fish feeds has more than likely accelerated their recent marketing. Nevertheless, Europe has a rigorous regulatory framework when it comes to GM crop cultivation. Indeed, some of the companies looking to grow transgenic n-3 LC-PUFA crops are focussing outside Europe in places such as the US or Canada where GM crop authorisation can be granted more quickly, often by up to 15–20 months as compared to Europe (Lucht 2015).

Perhaps the greatest challenge in getting GM products to market, including animal feed, is consumer acceptance, particularly in some European countries. It is thought that around 90% of soybeans traded globally contain GM material and, as such, a large proportion of animal feed available in the EU already contains GM components (Lucht 2015; Van Eenennaam and Young 2014). The EU’s GM Food and Feed Regulation (EC) No. 1829/2003 currently states that products derived from an animal reared on feeds containing GM ingredients do not need to be labelled as such. Therefore, while oils from transgenic plants would have to be labelled as GM if sold directly to humans, salmon reared on feeds containing the oils would not. Informing and educating the public could potentially increase acceptance. For example, those that feel more informed about science and GM foods, particularly when the potential benefits are pointed out such as increased omega-3 content or sustainability, are more likely to support GM foods (Desaint and Varbanova 2013; Lucht 2015).

## Conclusion

The need to produce entirely novel n-3 LC-PUFA sources for use in aquaculture is greater than ever with the main finite marine resource, fish oil, being spread thinner as the farmed fish industry grows to meet demand from an increasing global population. It is evident that both microalgal and GM technologies possess the capabilities to fulfil some, if not all, of the necessary requirements to augment fish oil use in aquafeeds. It is likely different sectors of the aquaculture industry will favour one product over the other, most likely depending upon production volumes, economics, consumer acceptability or regulatory issues. However, the availability of such options should prevent further decline in n-3 LC-PUFA levels in aquaculture and eventually lead to these beneficial fatty acids being restored to previous levels to ensure that farmed fish will remain a product of high nutritional value.
